# Geniposide Protects Primary Cortical Neurons against Oligomeric Aβ_1-42_-Induced Neurotoxicity through a Mitochondrial Pathway

**DOI:** 10.1371/journal.pone.0152551

**Published:** 2016-04-05

**Authors:** Chunhui Zhao, Cui Lv, Hang Li, Shijing Du, Xiaoli Liu, Zhi Li, Wenfeng Xin, Wensheng Zhang

**Affiliations:** 1 Beijing Area Major Laboratory of Protection and Utilization of Traditional Chinese Medicine, Beijing Normal University, Beijing, China; 2 Laboratory of Immunology for Environment and Health, Shandong Analysis and Test Center, Shandong Academy of science, Jinan, China; 3 College of Resources Science Technology, Beijing Normal University, Beijing, China; 4 Engineering Research Center of Natural Medicine, Ministry of Education, Beijing Normal University, Beijing, China; 5 Engineering Research Center of Sanqi Biotechnology and Pharmaceutical, Yun Nan Province, Kunming, China; Karolinska Institutet, SWEDEN

## Abstract

Mitochondrial dysfunction plays a key role in the progression of Alzheimer’s disease (AD). The accumulation of amyloid-beta peptide (Aβ) in the brains of AD patients is thought to be closely related to neuronal mitochondrial dysfunction and oxidative stress. Therefore, protecting mitochondria from Aβ-induced neurotoxicity is an effective strategy for AD therapeutics. In a previous study, we found that geniposide, a pharmacologically active compound purified from gardenia fruit, has protective effects on oxidative stress and mitochondrial dysfunction in AD transgenic mouse models. However, whether geniposide has a protective effect on Aβ-induced neuronal dysfunction remains unknown. In the present study, we demonstrate that geniposide protects cultured primary cortical neurons from Aβ-mediated mitochondrial dysfunction by recovering ATP generation, mitochondrial membrane potential (MMP), and cytochrome c oxidase (CcO) and caspase 3/9 activity; by reducing ROS production and cytochrome c leakage; as well as by inhibiting apoptosis. These findings suggest that geniposide may attenuate Aβ-induced neuronal injury by inhibiting mitochondrial dysfunction and oxidative stress.

## Introduction

Alzheimer’s disease (AD) is a progressive neurodegenerative disorder characterized by the clinical manifestation of severe memory impairment, cognitive deficits and personality changes [1–5]. The pathologic characteristics of AD are amyloid-beta peptide (Aβ) deposition, neurofibrillary tangle (NFT) formation and neuronal loss [6, 7].

To meet the high energy demands of brain, mitochondria play an important role in central nervous system neurons. Moreover, it is reported that molecular indices of mitochondrial dysfunction occur early in AD and worsen with progression [8–11]. Recently, increasing evidence suggested that Aβ is the key factor contributing to mitochondrial dysfunction [7, 12–14]. There are several ways for Aβ to damage neuronal mitochondria, cell membrane, cellular matrix, intermembrane space, outer/inner mitochondrial membrane, and the matrix. Among them, a transmembrane receptor of the immunoglobulin super family, receptor for advanced glycation end products (RAGE) play an important role in mediating Aβ-induced mitochondrial dysfunction [15].

The binding of Aβ and membranal RAGE can activate NADPH oxidase which is one of the major source of ROS [16]. Over-producted ROS may cause mitochondrial dysfunction due to lowered ETC enzyme activities. In addition, RAGE mediate the tranlocation of Aβ across the cytomembrane from extracellular to intracellular [17]. Aβ and amyloid precursor protein (APP) proteins were found localizing and accumulating in the mitochondrial membrane and matrix, which directly cause a series of terrible outcomes, such as opening the mitochondrial permeability transition pore (mtPTP), disrupting the electron transport chain (ETC), and reducing cytochrome c oxidase (CcO) activity. These problems may result in loss of mitochondrial membrane potential (MMP), decreased ATP production, increased levels of reactive oxygen species (ROS) and impaired calcium homeostasis, leading to neuronal apoptosis and the aggravating pathology changes of AD [13, 14, 18–23]. Therefore, protecting mitochondria against the structural and functional damage of Aβ is an effective strategy for AD therapeutics.

Geniposide is an iridoid glucoside isolated from the gardenia fruit (Gardenia jasminoides Ellis, Rubiaceae) and has diverse pharmacological capabilities including anti-inflammatory [24], anti-oxidation [25] and anti-tumour [26] effects, as well as neurotrophic and neuroprotective properties. As we previously reported, geniposide could inhibit both oxidative stress and mitochondrial dysfunction and could improve cognition in an Alzheimer’s disease mouse model [27, 28]. Furthermore, our studies suggest that geniposide inhibits the interaction of Aβ and RAGE [29]. In addition, the ability to cross the blood-brain barrier (BBB) allows geniposide to enter the central nervous system and act on neurons or other brain cells [30, 31]. Therefore, we wonder whether geniposide has any neuroprotective effect on Aβ-induced mitochondrial dysfunction. In the present study, by cultured primary cortical neurons, we investigated the effects of geniposide on oligomeric Aβ-induced mitochondrial dysfunction related to neurotoxicity and apoptosis. Our results show that geniposide treatment significantly attenuates neuronal apoptosis by ameliorating mitochondrial dysfunction.

## Material and Methods

### Reagents

**Fig 1:**
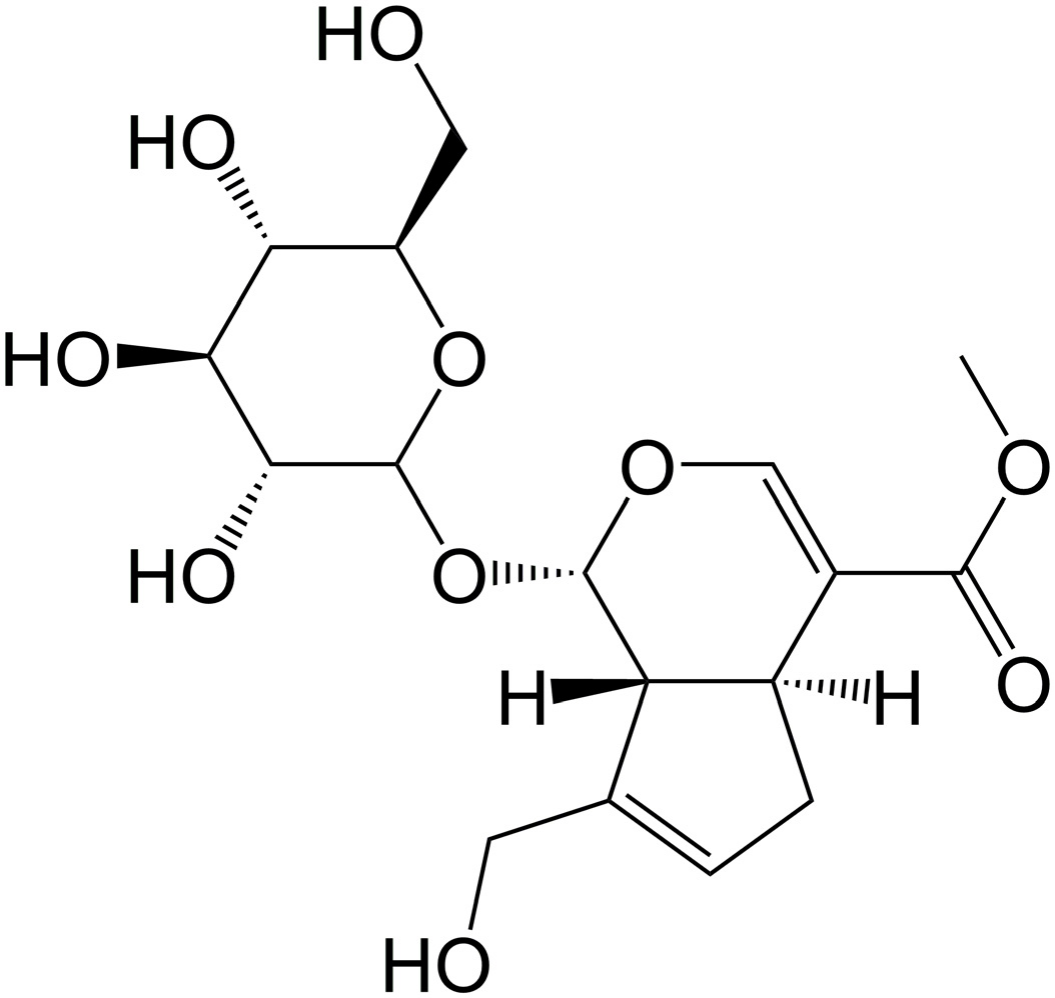
Chemical structure of geniposide.

Geniposide (Purity: > 98%, Fig 1) was purchased from the National Institute for the Control of Pharmaceutical and Biological Products (Beijing, China) and was free of endotoxin. 1,1,1,3,3,3-Hexafluoro-2-propanal (HFIP), 3-(4, 5-dimethylthiazol-2-yl)-2,5-diphenyltetrazo-lium (MTT) and penicillin/streptomycin were from Sigma (St. Louis, MO, USA). Fetal bovine serum (FBS), B27, and Neurobasal medium were from Gibco. Monoclonal mouse antibody against cytochrome c (1:1000 in PBST containing 0.1% Tween-20 and 5% skimmed milk, Cat# ab110325) [32] and cytochrome c oxidase (CcO, 1:500 in PBST containing 0.1% Tween-20 and 5% skimmed milk, Cat# ab110259) [33] were purchased from Abcam (Cambridge, UK). Monoclonal mouse antibodies against Bcl-2 (B-cell lymphoma-2, 1:500 in PBST containing 0.1% Tween-20 and 5% skimmed milk, Cat# SC-509) [34], Bax (Bcl-2 associated X protein, 1:500 in PBST containing 0.1% Tween-20 and 5% skimmed milk, Cat# SC-20067) [35] and β-actin (1:2000 in PBST containing 0.1% Tween-20 and 5% skimmed milk, Cat# SC-47778) [36] were purchased from Santa Cruz (CA, USA). Monoclonal goat anti-mouse (1:10000 in PBST containing 0.1% Tween-20 and 5% skimmed milk) secondary antibody were provided by GE Healthcare (Buckinghamshire, UK). The kits employed to isolate the mitochondria and to detect the levels of MDA, caspase 3/9 and the degree of mitochondrial oxidative damage were purchased from Shanghai GENMED Pharmaceutical Technology Co., Ltd. (Shanghai, China). The kits employed to detect the level of CcO activity and ATP were purchased from Sigma (St. Louis, MO, USA) and Roche (Indianapolis, USA), respectively. 2’,7’-Dichlorodihydrofluorescein diacetate (DCFH-DA) was purchased from Sigma Chemical Co. (St. Louis, MO, USA). All other chemicals were reagent grade.

### Oligomer Aβ preparation

Oligomeric Aβ_1–42_ was prepared from commercially available synthetic peptides (Sigma Chemical Co., St. Louis, MO, USA) as described [37, 38]. Briefly, lyophilized peptide was resuspended in cold 1,1,1,3,3,3-hexafluoro-2-propanol (HFIP) at a concentration of 1 mg/mL and aliquoted into microcentrifuge tubes to obtain 0.1 mg stocks quickly. The stocks were stored at room temperature and protected from light for 2–24 hours before removing HFIP under gentle vacuum, leaving a thin transparent film of peptides at the internal surface of the tube, and then stored at -20°C. For the aggregation protocols, HFIP-treated peptide was dissolved in anhydrous dimethyl sulphoxide (DMSO) at 5 mM and diluted to 100 μM in PBS, pH 7.4. Diluted peptides were then incubated at 4°C for 24 hours to obtain oligomeric Aβ_1–42_.

### Cortical neuron culture and treatment

All animal procedures were carried out in accordance with the National Institute of Health Guide for the Care and Use of Laboratory Animals (NIH Publications No. 80–23, revised 1996), and the Beijing Normal University Laboratory Animals Care and Use Committee approved the study protocol. Animals were sacrificed by cervical dislocation, All efforts were made to minimize the number of animals used and their suffering.

Male and female C57BL/6 mice, aged 6–8 weeks and purchased from Beijing HFK Bio-Technology. Co. Ltd., were housed 2 per cage at an ambient temperature of 23 ± 1°C and relative humidity of 55 ± 5% under a 12 h light/dark cycle. Animals had free access to water and food.

Primary cortical neurons were prepared from the cortices of 1-day-old newborn pups. Briefly, the cortices were dissected in cold PBS. Tissues were collected and washed in PBS, and 0.05% (v/v) trypsin was added for digestion at 37°C for 15 minutes. The digestion was stopped by the addition of fetal bovine serum to a final concentration of 10% (v/v). Cells were collected by centrifugation at 800 x g for 10 minutes to remove the PBS and were resuspended in Neurobasal medium supplemented with 2% (v/v) B27.

Cells were plated onto pre-coated poly-D-lysine (10 μg/ml) 6-, 24- or 96-well plates (~ 5×10^4^ cells/ml) for different analyses. Cells were cultured at 37°C and 5% CO_2_ until use. Half of the initial medium was removed at day 2 and replaced with fresh medium. After seven days, the medium was replaced with Neurobasal medium without serum and phenol red (which affects the aggregation of Aβ). To probe the cytotoxicity of Aβ aggregates, neurons were incubated with the indicated concentrations of Aβ_1–42_ aggregates for 24 h, and cell viability was determined using the MTT assay. To explore the protection of geniposide on Aβ_1–42_ aggregate-treated neurons, primary cultured cortical neurons were pre-incubated for 1 h in the absence or presence of geniposide (2.5, 5, and 10 μM) before the addition of oligomeric Aβ_1–42_ (5 μM) for 24 h.

### Identification of cultured cortical neurons

Neurons plated on Greiner CELLview dish were rinsed with PBS three times and then fixed in 4% paraformaldehyde for 25 min at 4°C. Fixed cells were incubated in 0.1% (v/v) sodium citrate contain 0.1% (v/v) TritonX-100 for 2 min on ice, and then neurons were washed twice with PBS and incubated with 300 μl Anti-NeuN (1:500 in PBS containing 10% goat serum, Cat# ab177487) monoclonal primary rabbit antibody for 2 hr at 37°C. An Alexa Fluor^®^ 594-conjugated goat anti-rabbit IgG (1/1000) was used as the secondary antibody. DAPI (4',6-diamidino-2-phenylindole) was added for 10 min at room temperature followed by PBS washing to fluorescently label nuclei. Samples were photographed using a fluorescence microscope (LEICA DMI3000, Japan) and analysed using the Leica application suite. Image J software was used for counting cells. Three independent experiments were performed, and cells were counted in three fields per well. Data were expressed as the ratio of NeuN labelled neurons to total neurons.

### MTT

To explore the cytotoxicity of oligomeric Aβ_1–42_ and geniposide, as well as the effect of geniposide on cell viability induced by oligomeric Aβ_1–42_, cell viability was determined by the MTT colourimetric assay after the cells were treated. Cells were incubated for 4 h at 37°C with MTT (0.5 mg/ml final concentration) and dissolved in fresh complete medium in which metabolically active cells reduced the dye to purple formazan. Formazan crystals were dissolved with DMSO, and the absorbance was measured on a microplate reader (Thermo Multiskan MK3, USA) using a reference wavelength of 630 nm and a test wavelength of 570 nm.

### Assessment of Apoptosis

Apoptosis of Aβ_1–42_-treated neurons was detected with the In Situ Cell Death Detection Kit, Fluorescein (Roche, USA) according to the manufacturer's protocol. Briefly, neurons were rinsed with PBS three times and then fixed in 4% paraformaldehyde for 25 min at 4°C. Fixed cells were incubated in 0.1% (v/v) sodium citrate contain 0.1% (v/v) TritonX-100 for 2 min on ice, and then neurons were washed twice with PBS and incubated with 50 μl TUNEL reaction mixture for 60 min at 37°C in a humidified chamber in the dark. DAPI was added for 10 min at room temperature followed by PBS washing to fluorescently label nuclei. Negative controls were performed by adding 50 μl label solution at the same time. Samples were photographed using a fluorescence microscope (LEICA DMI3000, Japan) and analysed using the Leica application suite. Image J software was used for counting cells. Three independent experiments were performed, and cells were counted in three fields per well. Data were expressed as the ratio of apoptotic neurons to total neurons.

### ROS Measurement

Reactive oxygen species (ROS) were measured with 2’,7’-dichlorodihydrofluorescein diacetate (DCFH-DA), which detects most ROS including hydrogen peroxide, peroxyl radicals, and peroxynitrite anions. The cell-permeable DCFH-DA crosses into the cytoplasm, where it is de-esterified by cellular esterase resulting in DCFH. In turn, DCFH is converted upon oxidation to the highly fluorescent DCF. By measuring the fluorescence, we were able to quantify the levels of ROS. After incubation with Aβ_1–42_ for varying lengths of time, neurons were washed with PBS and incubated with 10 μM non-fluorescent DCFH-DA for 30 min at 37°C in the incubator with 5% CO_2_. The fluorescence intensity of DCF was measured with 485 nm excitation and 528 nm emission on a fluorescence microplate reader (BioTek Senergy HT, Vermont, USA). ROS levels are presented as arbitrary fluorescence units (AFU).

### Measurement of MDA

The levels of malondialdehyde (MDA) were measured using commercially available assay kits according to the instructions recommended by the manufacturers. In brief, cells were washed with PBS and collected with the assay buffer provided in the kits and then further homogenized by sonication using 5 pulses of 30 seconds each on ice. The homogenates were centrifuged at 3000 x g for 20 min at 4°C to obtain the supernatant. All protein concentrations of cell homogenate samples were determined by the BCA method, and then samples were diluted with the appropriate buffer solution to determine the level of MDA. The optical density (OD) of the microplate was read at 532 nm.

### Mitochondria Membrane Potential

Loss of mitochondrial membrane potential was determined using the JC-1 mitochondrial transmembrane potential detection kit (Cell Technology, Inc.) according to the manufacturer’s instructions. Briefly, cells were plated and cultured in glass-bottom dishes with four chambers. After cells were washed with PBS, 0.5 mL of 1× JC-1 reagent was added to each chamber. Then, cells were incubated at 37°C in a 5% CO_2_ incubator for 20 min. The cells were then washed with assay buffer (1×) and measured with a laser-scanning confocal microscope. Red fluorescence (excitation 550 nm, emission 600 nm) and green fluorescence (excitation 485 nm, emission 535 nm) were measured. The ratio of red to green fluorescence, an indicator for membrane potential, was determined. The ratio was decreased in dead cells and in cells undergoing apoptosis compared to healthy cells.

### Measurement of Cytochrome c Oxidase (CcO) activity

CcO activity was measured according to a previously published method [39]. Briefly, neuronal cultures in 6-well plates were washed with ice-cold PBS, and cells were harvested, centrifuged, and suspended in 50 μl of isolation buffer containing 250 mM sucrose, 20 mM HEPES, pH 7.2 and 1 mM EDTA. Cell suspensions (containing ~3–4 mg of protein/ml) were added to a cuvette containing 0.95 ml of 1× assay buffer (10 mM Tris-HCl, pH 7.0, and 120 mM KCl), and the reaction volume was brought to 1.05 ml with 1× enzyme dilution buffer (10 mM Tris-HCl, pH 7.0). The reaction was started by the addition of 50 μl of ferrocytochrome c substrate solution (0.22 mM) (from Sigma). The change in absorbance of cytochrome c at 550 nm was measured using a UV-2450 spectrophotometer (Shimadzu Company, JAPAN). The reading was recorded every 10 seconds during the first 3 min. Background levels were measured without cell suspensions.

### ATP Measurement

ATP levels were determined using a commercial kit (Roche, Indianapolis, USA). Briefly, neurons were treated with geniposide and/or oligomeric Aβ_1–42_ for different lengths of time. Neurons were harvested, centrifuged and diluted at a concentration of 1 × 10^4^ cells/ml. The same volume of cell lysis reagent was added to the samples and then incubated for 5 min at 25°C. An appropriate volume of luciferase reagents was added to the samples, and readings were recorded consecutively from 1 to 10 seconds with an interval of 1 second using the fluorescence microplate reader (BioTek Senergy HT, Vermont, USA). The changes between different groups were compared.

#### Isolation of mitochondrial and cytosolic fractions

The Mitochondrial Fractionation Kit (Active Motif, Carlsbad, CA, USA) was used to isolate mitochondrial and cytosolic fractions from cells according to the manufacturer’s instructions. Briefly, the treated neurons were scraped and centrifuged twice at 600 × g for 5 min. Ice-cold 1× cytosolic buffer was added, and the cell pellet was resuspended and incubated on ice for 15 min. Cells were homogenized, and the lysate was centrifuged twice at 800 × g for 20 min. The resultant supernatant contained the cytosol, including mitochondria; the supernatant was centrifuged at 10,000 × g for 20 min to pellet the mitochondria. The mitochondrial pellet was washed and centrifuged with 1× cytosolic buffer at 10,000 × g for 10 min and then lysed by adding Complete Mitochondria Buffer from the kit, followed by incubation on ice for 15 min, the result of which was the mitochondrial fraction. At the same time, the supernatant was centrifuged at 16,000 × g for 25 min. The centrifuged supernatant was the cytosolic fraction. The protein concentration was measured by the BCA method. After isolation of the mitochondria and cytosolic fractions, the western blot assay was used to measure the level of cytochrome c.

### Caspase-3/9 activity assay

Caspase-3 and -9 activities were independently assessed using the Caspase-3 Assay Kit and the Caspase-9 Assay Kit according to the instructions recommended by the manufacturers. Caspase-3 activity was assessed by reading excitation at 380 nm and emission at 460 nm in a microplate fluorometer (BioTek Senergy HT, Vermont, USA). Caspase-9 activity was assessed with excitation at 485 nm and emission at 528 nm.

### Western blot analysis

Neurons were prepared in RIPA lysis buffer containing a cocktail of complete protease inhibitors (Roche Diagnostics). After the determination of protein concentration by the BCA method, proteins were analysed by western blot according to standard protocols. In brief, equal amounts of protein were loaded and separated by SDS-PAGE and transferred to nitrocellulose membranes (Millipore). Membranes were blocked in PBST buffer (20 mM Tris-HCl, 150 mM NaCl, 0.1% Tween-20) containing 5% skimmed milk (Santa Cruz) for 1 h at room temperature on a rotary shaker and then incubated and gently shaken overnight (at 4°C) with primary antibodies against CcO (1:500), cytochrome c (1:1000), Bax (1:500), Bcl-2 (1:500) or β-actin (1:2000) in PBST containing 5% skimmed milk; this was followed by incubation with the corresponding secondary antibody for 1 h at room temperature. The blots were washed twice with PBST and developed with an Infrared Imaging System (Odyssey). The intensity of blots was analysed and compared using the NIH Image J program.

### Statistical analyses

The results were processed for statistical analysis using SPSS (version 13.0 for Windows). The results are presented as the mean ± SEM. Biochemical data were analysed using one-way ANOVA followed by independent single t-tests. The significance level was set at p < 0.05.

## Results

### Identification of cultured cortical neurons

**Fig 2:**
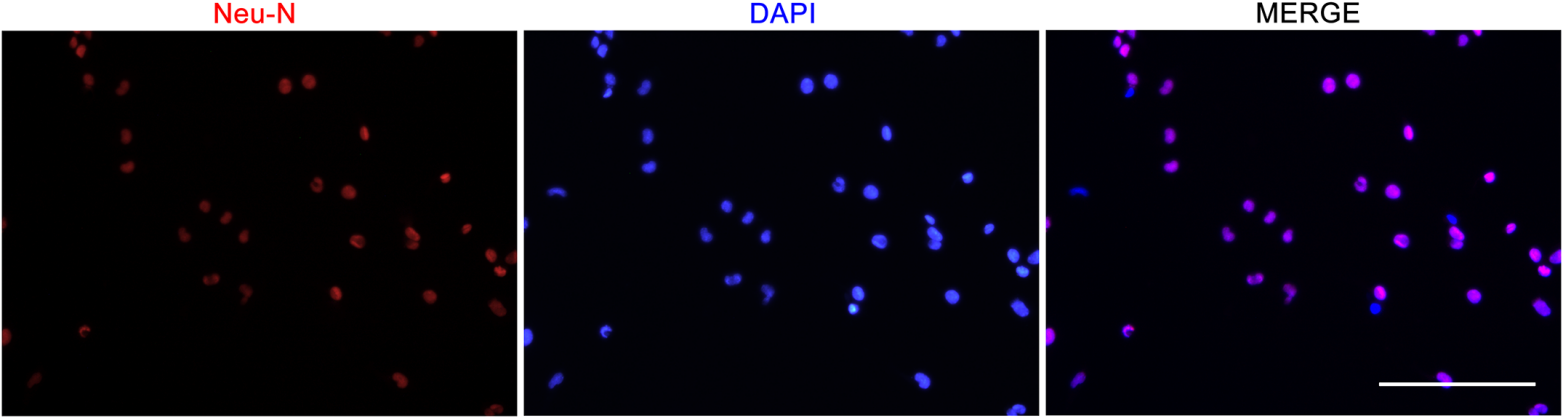
Immunocytochemsitry analysis of neurons labelling nuclei with NeuN. Neuronal nuclei were labelled with specific neuronal marker-NeuN (red), while all cells’ nuclei were stained in blue.

In the described culture condition, the purity of cultured mouse cortical neuron was 93.00% ± 1.23% at 7 days in vitro (Fig 2).

### Geniposide protected against Aβ_1-42_-induced cell viability loss in neurons

**Fig 3:**
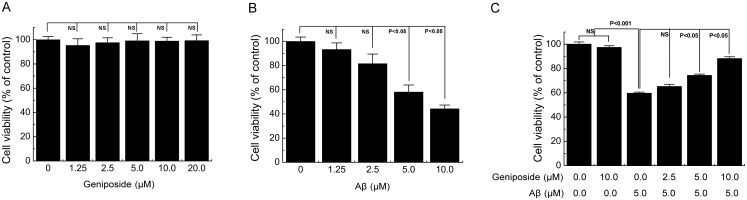
Geniposide prevents the cytotoxicity of oligomeric Aβ_1–42_ in neurons. A, Effects of geniposide on the cell viability in neurons. Cells were treated with the indicated doses of geniposide for 24 h, and the cell viability was determined with MTT. B, Effects of oligomeric Aβ_1–42_ on the cell viability in the primary cultured cortical neurons. After cells were treated with the indicated doses of Aβ_1–42_ for 24 h, the cell viability was determined with MTT. C, Geniposide increases the cell viability of neurons induced by Aβ_1–42_. Cells were pre-incubated with geniposide at 2.5–10 μM for 24h, and then exposed to oligomeric Aβ_1–42_ at 5.0 μM for 24 h in the presence and absence of geniposide. The cell viability was measured by MTT. NS: non significance. N = 6 per group of cells. Studies were repeated four times and data were expressed as means ± SEM of percentage of vehicle-treated cells.

First, the neurons were treated with various concentrations (1.25, 2.5, 5, 10, and 20 μM) of geniposide for 24 h to determine whether geniposide is toxic to neurons. Cell viability was assessed by the MTT assay. The results showed that geniposide exhibited no significant neurotoxicity to the neurons (Fig 3A). Next, to determine the appropriate toxic concentration of Aβ_1–42_, the neurons were incubated with four different concentrations (1.25, 2.5, 5, and 10 μM) of Aβ_1–42_ for 24 h, and the cell viability was assessed by the MTT assay. The results showed that oligomeric Aβ_1–42_ peptides induced neurotoxicity in a dose-dependent manner (Fig 3B). Given these results, 5 μM Aβ_1–42_ was selected to incubate the primary cortical neurons for 24 h in the further experiments. Third, we detected the effect of geniposide on Aβ_1-42_-induced cell viability loss in neurons. Neurons were pre-treated for 24 h with various concentrations (2.5, 5, and 10 μM) of geniposide, followed by treatment with 5 μM Aβ_1–42_ for 24 h. As shown in Fig 3C, cell viability decreased to 59.59% ± 0.94% after exposure to 5 μM Aβ_1–42_ for 24 h, and pre-treatment with geniposide (2.5, 5, and 10 μM) for 24 h could enhance cell viability to 65.14% ± 1.81%, 74.29% ± 1.10%, and 88.12% ± 1.52% (Fig 3C), respectively. Therefore, 2.5, 5, and 10 μM geniposide were selected for all of the subsequent experiments.

### Geniposide suppressed Aβ_1-42_-induced apoptosis in neurons

**Fig 4:**
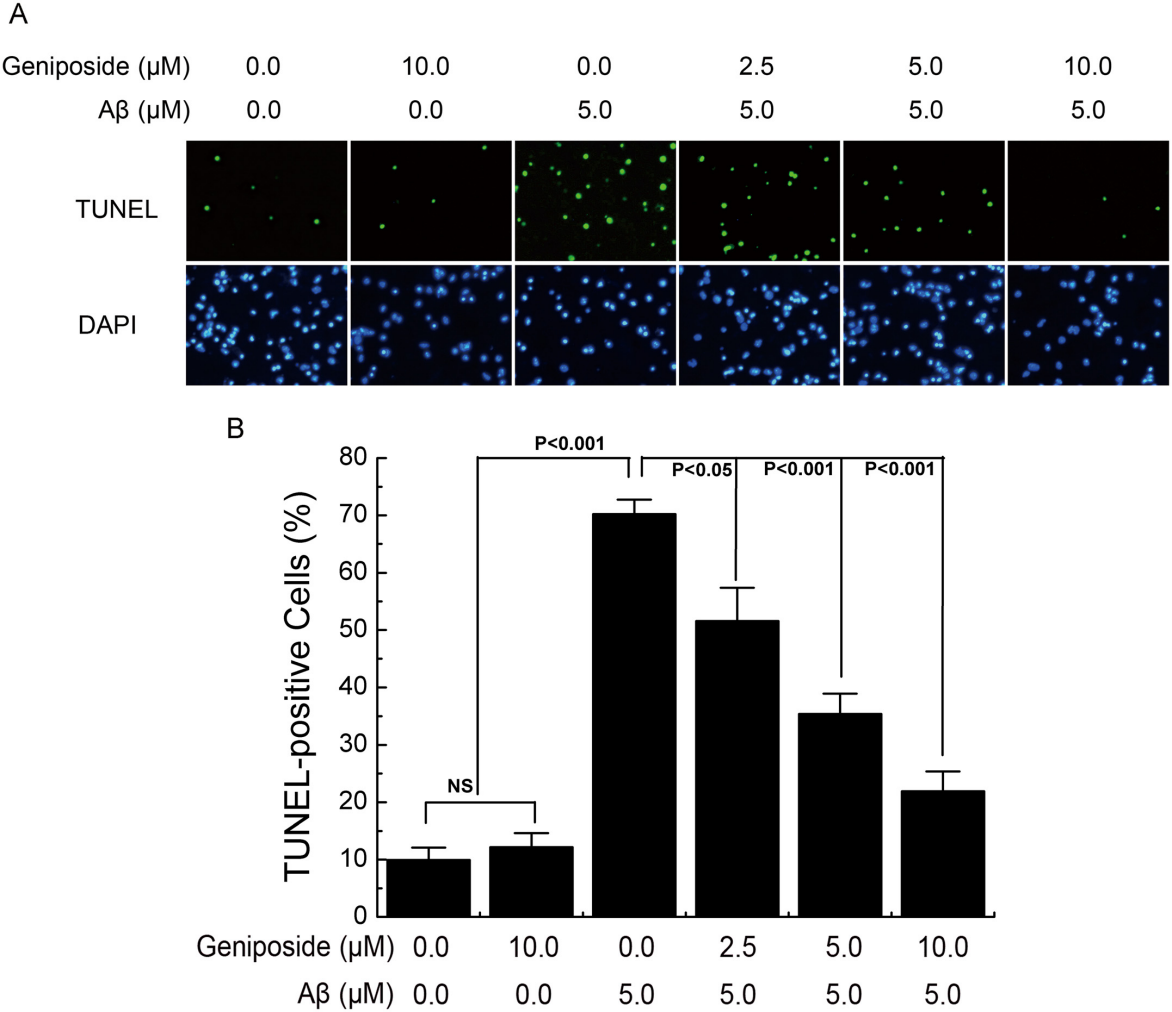
The effect of geniposide on Aβ_1-42_-induced apoptosis. A, Photomicrographs of TUNEL and DAPI fluorescence staining. Neurons were cultured in the presence of 5 μM oligomeric Aβ_1–42_ and in the presence and absence of geniposide (2.5 μM, 5 μM, 10 μM) for 24 h. TUNEL-positive cells were stained in green while all cells’ nuclei were stained in blue. B, Quantitative analysis the ratio of TUNEL-positive cells to total neuron number. The ratio of TUNEL-labelled neurons was significantly increased in neurons treated with oligomeric Aβ_1–42_ for 24 hours while the geniposide treatment dramatically reduced the Aβ-induced TUNEL-positive cells in a dose-dependent manner. NS: non significance. N = 6 per group of cells. Studies were repeated thrice and data were expressed as mean ± SEM of percentage of vehicle-treated cells.

To investigate the protective effect of geniposide on Aβ-induced apoptosis, we applied TUNEL staining to detect the number of apoptotic neurons. Primary cultured neurons were pre-incubated for 24 h in the absence or presence of geniposide (2.5, 5 and 10 μM) before addition of oligomeric Aβ_1–42_ (5 μM) for 24 h and then stained by TUNEL and analysed under a fluorescence microscope. As shown in Fig 4A, TUNEL-labelled cells (green) were significantly increased in Aβ-treated neurons while precious little numbers of TUNEL-positive neurons were found in the vehicle-treated group. Treatment with geniposide significantly decreased the number of TUNEL-labelled cells in a dose-dependent manner. Statistical result of TUNEL staining (Fig 4B) suggested that Aβ observably increased the apoptosis rate (70.21%, p<0.001). By contrast, different concentrations of geniposide (2.5, 5 and 10 μM) reversed the Aβ-induced apoptosis(51.56%, P<0.05; 35.40%, P<0.001; 21.91%, P<0.001). Treatment with geniposide alone at 10μM showed no evident effect on the apoptosis rate, indicating that geniposide is not harmful to the neurons.

### Geniposide attenuated Aβ_1-42_-induced ROS production in neurons

**Fig 5:**
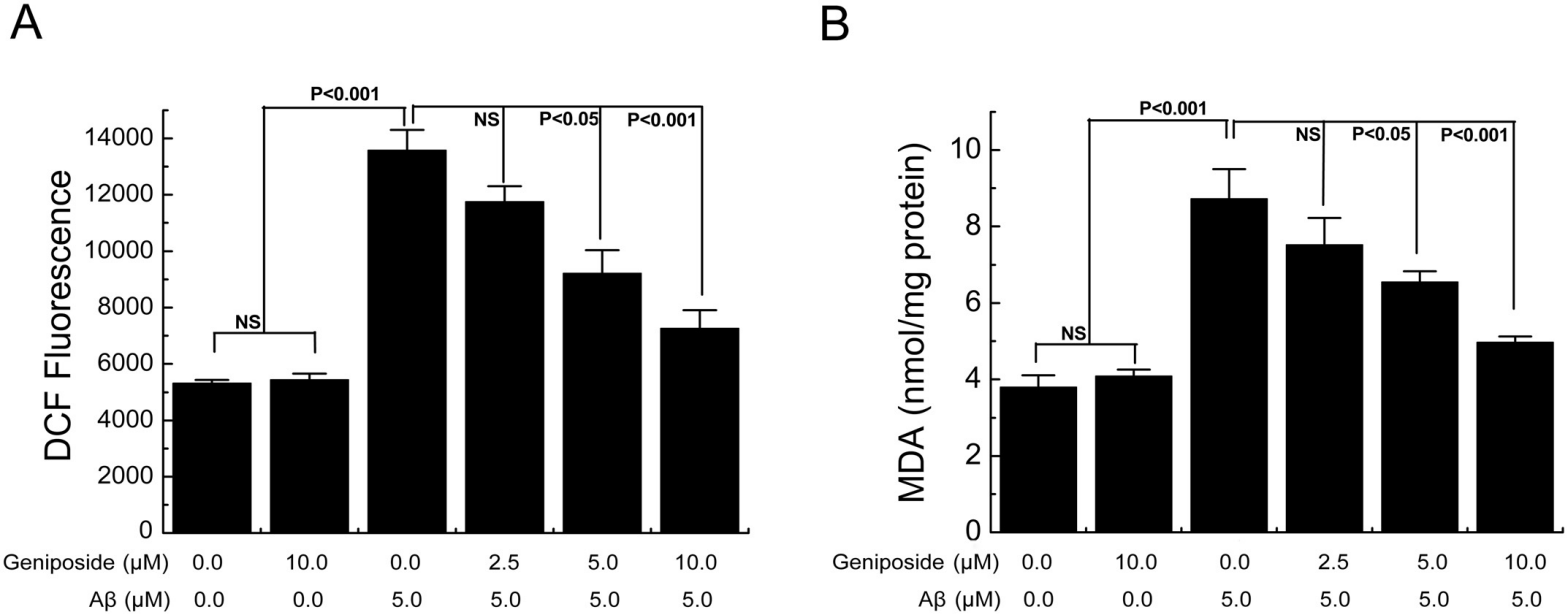
Effects of geniposide on the ROS and MDA levels in primary cultured cortical neurons. The levels of DCF fluorescence intensity (an indicator of ROS generation (A)) and MDA production (B) were significantly elevated in Aβ_1-42_-treated neurons compared to vehicle-treated neurons, and partially restored in the geniposide-treated neurons in a dose-dependent manner. NS: non significance. N = 6 per group of cells. Studies were repeated four times and data were expressed as mean ± SEM of percentage of vehicle-treated cells.

Mitochondria are major sources for the generation and accumulation of reactive oxygen species (ROS), and mitochondrial dysfunction causes increased radical production. To examine whether geniposide could suppress cell apoptosis via its antioxidant effects, we determined the effect of geniposide on Aβ_1-42_-induced oxidative stress. The levels of intracellular ROS and a marker of lipid peroxidation (malondialdehyde, MDA) were examined simultaneously, both of which were significantly increased in neurons exposed to Aβ_1–42_. The level of ROS in Aβ_1-42_-treated neurons was significantly increased (p<0.001) compared with the control group. However, pre-treatment with geniposide (5 and 10 μM) decreased the level of ROS compared with the Aβ_1-42_-treated group (p<0.05, p<0.001) (Fig 5A). The level of MDA in Aβ_1-42_-treated neurons was significantly increased (p<0.001) compared with the control group. However, pre-treatment with geniposide (5 and 10 μM) could decrease the level of MDA (p<0.05, p<0.001) (Fig 5B). These results suggest that geniposide plays a protective role against oxidative stress by decreasing the generation of ROS and MDA, which suggests that the antioxidant effects of geniposide may be involved in the neuroprotection mechanism.

### Geniposide protected against Aβ_1-42_-induced mitochondrial dysfunction

**Fig 6:**
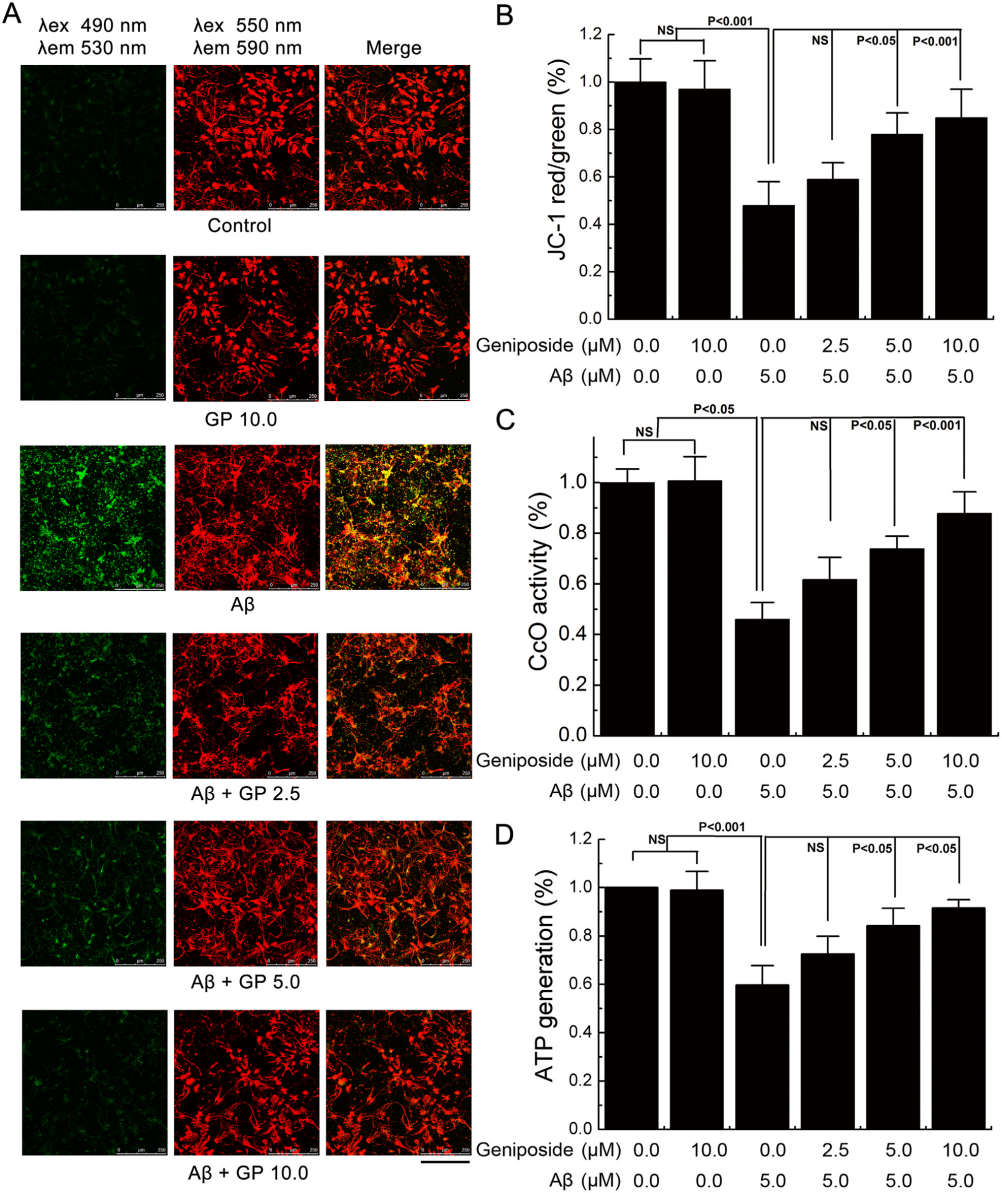
The effects of geniposide on Aβ_1-42_-induced mitochondrial abnormalities. A, Mitochondrial membrane potential. Cells were labeled with JC-1 dye and analyzed by laser-scanning confocal microscope per the manufacturer’s protocol. In the live non-apoptotic cells, the mitochondria appearred following aggregation of JC-1 reagent. In the apoptotic cells, the dye remains in its monomeric form and appears green. Scalebar = 250 μm. B, Quantitative analysis of the Δψm among groups. The ratio of red to green fluorescence in A was used as the indicator for mitochondrial membrane potential. C, The ATP level was determined by fluorescence microplate reader. D, CcO activity was tested by spectrophotometer. NS: non significance. N = 6 per group of cells. Studies were repeated four times and data were expressed as mean ± SEM of percentage of vehicle-treated cells.

To examine whether geniposide could preserve mitochondrial function, mitochondrial membrane potential (ΔΨM), ATP levels and cytochrome c oxidase (CcO) were assessed. Neurons were pre-treated with varying doses of geniposide for 24 h and co-incubated with or without oligomeric Aβ_1–42_ for 24 h and then collected for further analysis. As shown in Fig 6A and 6B, a significant loss of ΔΨM was observed in neurons treated with Aβ_1–42_ (p<0.001). However, pre-treatment with varying doses of geniposide (2.5, 5, 10 μM) showed a dose-dependent increase in ΔΨM compared to neurons treated with Aβ_1–42_ alone.

Next, we measured ATP levels. Decreases in ATP levels correlated with the changes in ΔΨM. As shown in Fig 6C, a significant decrease in ATP levels (59.67% compared to control, p<0.001) was observed in neurons treated with Aβ_1–42_, which could be alleviated by geniposide treatment in a dose-dependent manner.

Third, we analysed mitochondrial respiratory function by measuring CcO activity. Defects in CcO activity have been shown in early stage AD brains, AD mouse models, and Aβ-treated neurons. As shown in Fig 6D, CcO activity was significantly decreased (46.02% compared to control, p<0.001) in Aβ_1-42_-treated neurons. However, geniposide(2.5, 5 and 10 μM) treatment significantly increased CcO activity in a dose-dependent manner(61.70%, NS; 73.89%, P<0.05; 87.82%, P<0.001).

### The effect of geniposide on mitochondrial pathways in Aβ_1-42_-induced neuronal apoptosis

To determine whether the protective effect of geniposide on Aβ_1-42_-induced mitochondrial dysfunction correlated with neurotoxicity, we assessed changes in cytochrome c release, caspase-3/9 activity and Bcl-2 and Bax expression.

**Fig 7:**
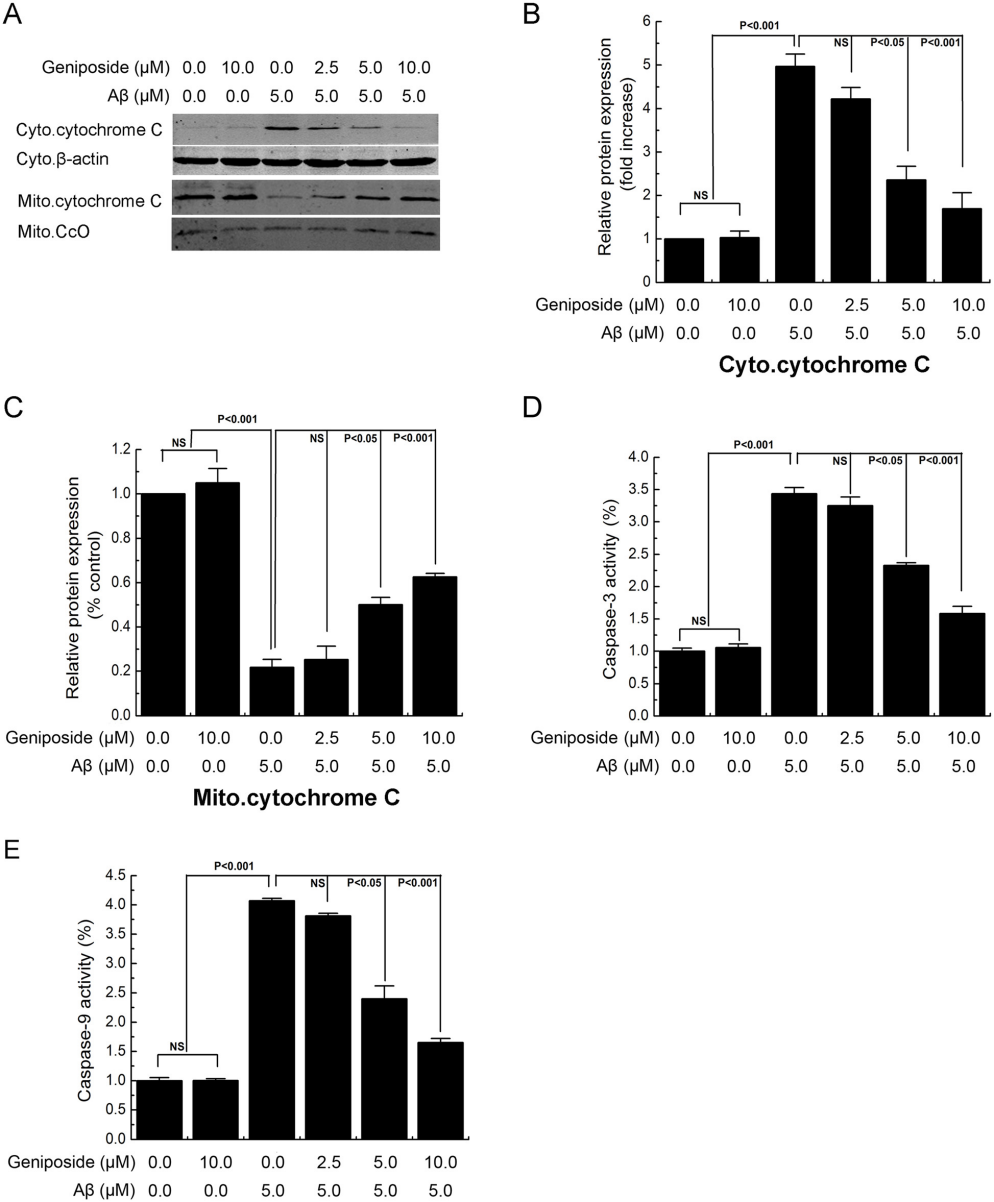
The effects of geniposide on Aβ_1-42_-induced release of mitochondrial cytochrome c and caspase-3/9 activity. Neurons were cultured in the presence of 5 μM oligomeric Aβ_1–42_ and geniposide (2.5 μM, 5 μM, 10 μM) for 24 h, harvested and the levels of specific proteins were assessed by western blot analysis (A). Levels of cytochrome c in the cytosolic fraction (B) or mitochondrial fractions (C), Geniposide attenuated mitochondrial cytochrome c releasing to cytosolic fraction in neurons induced by oligomeric Aβ_1–42_ at 5 μM. Caspase-3/9 activity was assessed by microplate fluorometer. The increased activity of caspase-3/9 was attenuated (d, e) by the present of geniposide in a dose-independent manner. NS: non significance. N = 6 per group of cells. Studies were repeated four times and data were expressed as mean ± SEM of percentage of vehicle-treated cells.

Mitochondrial dysfunction can also induce the release of cytochrome c from the mitochondria into the cytosol. Our results showed that Aβ_1–42_ caused a translocation of cytochrome c from the mitochondria into the cytosol after 24 h of treatment (Fig 7A, 7B and 7C). Pre-treatment with geniposide (2.5–10 μM) significantly attenuated the release of cytochrome c into the cytosol in a dose-dependent manner, showing that the mitochondrial dysfunction induced by Aβ_1–42_ was rescued by geniposide.

The release of cytochrome c from mitochondria can activate caspase-9 and caspase-3. Activation of either caspase can cleave poly ADP-ribose polymerase (PARP) and trigger chromosomal DNA fragmentation. The results (Fig 7D and 7E) showed that both caspase-3 and caspase-9 activities were increased by the 24 h application of Aβ_1–42_. Treatment with geniposide significantly inhibited caspase-9 and caspase-3 activities in a dose-dependent manner.

**Fig 8:**
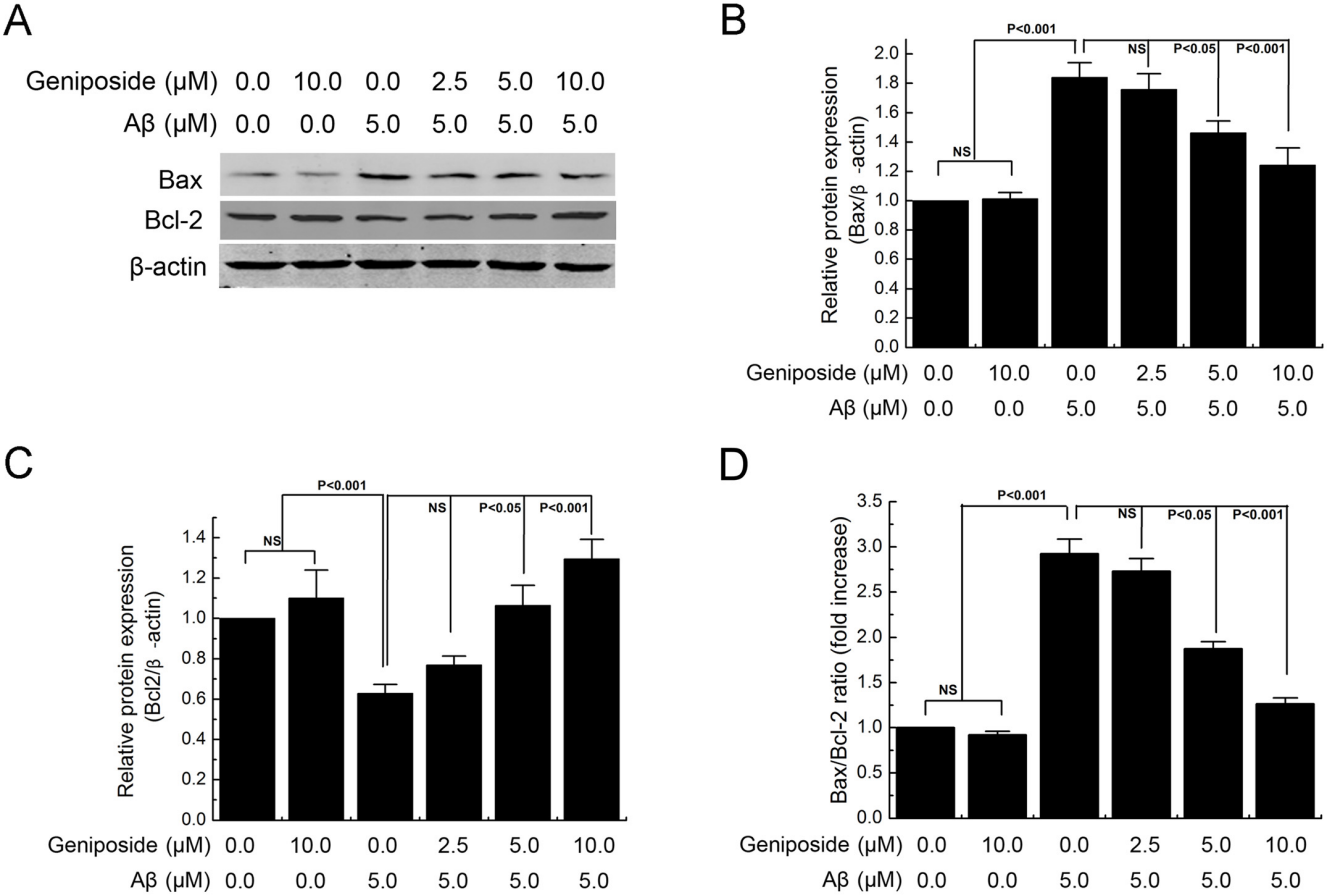
The effect of geniposide on Aβ_1-42_-induced Bax and Bcl-2 proteins abnormally expression level. Neurons were cultured in the presence of 5 μM oligomeric Aβ_1–42_ and geniposide (2.5 μM, 5 μM, 10 μM) for 24 h, harvested and the levels of specific proteins were assessed by immunoblot analysis (A). Pretreatment of primary cortical neurons with geniposide (2.5–10 μM) could upregulate Bcl-2 expression (B), downregulate Bax expression (C) and decrease the Bax/Bcl-2 ratio (D) induced by Aβ_1–42_. NS: non significance. N = 6 per group of cells. Studies were repeated four times and data were expressed as mean ± SEM of percentage of vehicle-treated cells.

Because translocation of the Bcl-2 family of proteins from the mitochondria to the cytosol is known to play a key role in mitochondrial-mediated apoptosis induced by a variety of apoptotic stimuli, western blot analysis was performed to assess the effect of geniposide on the expression of Bcl-2 (anti-apoptotic) and Bax (pro-apoptotic) proteins (Fig 8A). The results showed that treatment of primary cortical neurons with Aβ_1–42_ alone caused a significant decrease in the level of Bcl-2 (p<0.001) and a significant increase in the level of Bax (p<0.001) compared with the control group. However, pre-treatment of primary cortical neurons with geniposide (5 and 10 μM) could upregulate Bcl-2 expression (p<0.05, p<0.001) (Fig 8B) and downregulate Bax expression (p<0.05, p<0.001) (Fig 8C) induced by Aβ_1–42_. Thus, the Bax/Bcl-2 ratio significantly decreased (p<0.05, p<0.001) (Fig 8D).

## Discussion

Recent studies suggest that either intramitochondrial or extramitochondrial Aβ may directly cause mitochondrial dysfunction [15]. The dysfunction of mitochondria can lead to decreased ATP and MMP levels, increased ROS levels, the release of mitochondrial cytochrome c, elevated caspase-3 activity, and ultimately result in neuronal apoptosis.

According to the relevant articles [16, 40, 41] and our previous study [27–29], we hold the opinion that the binding of Aβ and membranal RAGE caused activation of NADPH oxidase and followed by generation of ROS is the major reason of mitochondrial dysfunction. In our previous studies [29], we proved that geniposide inhibits the interaction between Aβ and RAGE. So we thought geniposide may ameliorate mitochondrial dysfunction by inhibits the interaction between Aβ and membranal RAGE. Furthermore, Aβ can mediate its effect via other Aβ receptors. Murine PirB (paired immunoglobulin-like receptor B) and its human ortholog LilrB2 (leukocyte immunoglobulin-like receptor B2) act as receptors for oligomeric Aβ_1–42_ in human brains, mediated loss of synaptic plasticity and memory deficits [42]. Cellular prion protein (PrP^C^) and ephrin type B receptor 2 (EphB2) binds oligomeric Aβ and alter the function of NMDAR (NMDA receptor) in neurons, result in synaptic dysfunction [43–45]. However, whether geniposide has an effect on these receptors remains to be further investigated.

In addition, there is evidence to suggest that RAGE mediates transport of Aβ peptides across the cytomembrane from extracellular to intracellular [17, 46, 47]. Therefore, the application of geniposide may decrease the content of intracellular Aβ through inhibit the interaction of Aβ and RAGE. The accumulation of Aβ in the brain is a critical, early event that leads to neuronal dysfunction and memory impairment in AD pathogenesis. Mitochondria are the targets for both amyloid precursor protein (APP) and Aβ, which interact with several proteins inside mitochondria [15, 48]. Aβ may interact with respiratory chain complexes and affect the activity of several mitochondrial enzymes, such as the pyruvate dehydrogenase complex (PDH) and ketoglutarate dehydrogenase complex (KGDH) [49], as well as ETC enzyme complexes, especially complex IV (CcO), disrupting hydrogen transport across the mitochondrial intermembrane space and thus encouraging excessive MMP depolarization [50, 51].

One of the apoptotic factors, cytochrome c, transports electrons from complex III to IV along the ETC while protons are pumped from the matrix into the intermembrane space to form an electrochemical gradient (MMP) [15, 48]. Anti-apoptotic protein Bcl-2 and pro-apoptotic Bax are involved in the mitochondria-mediated apoptosis pathway. Bcl-2 binding to the mitochondrial membrane, competitive binding with Bax and the formation of the Bcl-2/Bax heterodimer lead to the closing of mtPTP and prevent the release of cytochrome c, while the binding of the Bax/Bax homodimer to the mitochondrial membrane and the formation of permeable channels lead to the release of cytochrome c, which couples with destroyed MMP and also activates the caspase-related apoptosis cascade reaction [52–55]. Our results showed that geniposide attenuated the level of increase of Bax/Bcl-2 and caspase-3/9 activity, the release of cytochrome c and the decrease in MMP induced by Aβ.

In addition, Recent studies shown that Aβ interaction with mitochondrial and plasma membrane voltage-dependent anion channel 1 (VDAC1) is involved in the extrinsic apoptotic pathway, and VDAC1 is overexpressed in post-mortem brains of Alzheimer disease (AD) patients, suggest that VDAC1 may serve as a potential target for AD treatment [56–58]. However, the mechanisms underlying it remains to be further investigated.

Plenty of evidence points to the fact that Aβ reduced the activity of the ETC enzyme complex IV (CcO) in isolated mitochondria or whole cells exposed to Aβ protein and in AD transgenic mice [59, 60]. Furthermore, CcO dysfunction may increase ROS production and reduce ATP levels, which in turn may impair the function of other mitochondrial enzymes by stalling electron transfer [50]. However, the protection mechanism of geniposide on CcO activity reduction needs further investigation.

AD brains experience significant neuron loss, which likely contributes to the progressive decline of memory and cognitive functions [51]. While some neuron loss is due to necrosis, the remainder is likely due to or invokes aspects of apoptosis, which is a tightly regulated form of programmed cell death [50]. DNA fragmentation, a common hallmark of apoptosis, is typically assessed by TUNEL staining. There was evidence that neurons in AD brains display increased DNA fragmentation compared to control brains [61, 62]. The present studies provide evidence of geniposide protection of neurons from Aβ-induced apoptosis.

In summary, the present study demonstrates that geniposide, the main active component of gardenia fruit, has protective effects against Aβ-mediated neurotoxicity in primary cultured mouse cortical neurons, via the recovery of ATP generation, MMP levels, CcO and caspase-3/9 activities, reduction of ROS production, as well as the inhibition of apoptosis. In addition, there is evidence demonstrating that geniposide could cross the BBB and produce protective properties in the brain [30]. Because AD has been considered to be a severe impairment of the brain, the detailed mechanisms underlying Aβ toxicity and the neuroprotective effects of geniposide required further investigation. Our results may contribute to provide support for potential applications of geniposide in AD treatment.
